# Novel insights into the modulation of the voltage-gated potassium channel K_V_1.3 activation gating by membrane ceramides

**DOI:** 10.1016/j.jlr.2024.100596

**Published:** 2024-07-15

**Authors:** Bence Cs. Szabo, Mate Szabo, Peter Nagy, Zoltan Varga, Gyorgy Panyi, Tamas Kovacs, Florina Zakany

**Affiliations:** Department of Biophysics and Cell Biology, Faculty of Medicine, University of Debrecen, Debrecen, Hungary

**Keywords:** ceramides, glycolipids, fluorescence microscopy, lipids, physical biochemistry, two-electrode voltage-clamp fluorometry, membrane biophysics, ion channels, K_V_1.3, lipid-protein interactions

## Abstract

Membrane lipids extensively modulate the activation gating of voltage-gated potassium channels (K_V_), however, much less is known about the mechanisms of ceramide and glucosylceramide actions including which structural element is the main intramolecular target and whether there is any contribution of indirect, membrane biophysics-related mechanisms to their actions. We used two-electrode voltage-clamp fluorometry capable of recording currents and fluorescence signals to simultaneously monitor movements of the pore domain (PD) and the voltage sensor domain (VSD) of the K_V_1.3 ion channel after attaching an MTS-TAMRA fluorophore to a cysteine introduced into the extracellular S3-S4 loop of the VSD. We observed rightward shifts in the conductance-voltage (G-V) relationship, slower current activation kinetics, and reduced current amplitudes in response to loading the membrane with C16-ceramide (Cer) or C16-glucosylceramide (GlcCer). When analyzing VSD movements, only Cer induced a rightward shift in the fluorescence signal-voltage (F-V) relationship and slowed fluorescence activation kinetics, whereas GlcCer exerted no such effects. These results point at a distinctive mechanism of action with Cer primarily targeting the VSD, while GlcCer only the PD of K_V_1.3. Using environment-sensitive probes and fluorescence-based approaches, we show that Cer and GlcCer similarly increase molecular order in the inner, hydrophobic regions of bilayers, however, Cer induces a robust molecular reorganization at the membrane-water interface. We propose that this unique ordering effect in the outermost membrane layer in which the main VSD rearrangement involving an outward sliding of the top of S4 occurs can explain the VSD targeting mechanism of Cer, which is unavailable for GlcCer.

Ion channels including voltage-gated potassium (K_V_) channels are embedded into the cell membrane and, therefore, complex conformational changes leading to their different conducting and non-conducting functional states occur through the permission and cooperativity of the surrounding bilayer. Consequently, these transitions and concomitantly the channel activity can be modulated by membrane lipids via the result of various direct ligand-like lipid-protein interactions and indirect effects, i.e. alterations in the biophysical properties of cellular membranes (1, 2). Such electrophysiological effects and the potential mechanisms of actions have been extensively documented for certain lipids, such as cholesterol (2, 3), phosphoinositides (4, 5, 6, 7), and polyunsaturated fatty acids (8, 9) acting on K_V_ channels as well, in which both direct actions and indirect membrane biophysical effects have been proposed to mediate these complex lipid-induced effects. Contrastingly, modulation of K_V_ ion channel activities by membrane incorporating ceramides and glucosylceramides is rather scarcely known in spite of the fact that elevated levels of these lipids are substantially linked to the pathomechanism of various human diseases including lysosomal storage disorders (10, 11), neurodegenerative (12) and metabolic diseases (13, 14).

K_V_1.3, a channel with essential physiological and pathophysiological roles in lymphocytes (15), macrophages (15), and microglia (16), is characterized by structural properties and gating mechanisms prototypical for other members of the K_V_ superfamily (17). K_V_1.3, just like other K_V_ channels, comprises four alpha subunits held together by noncovalent bonds, each containing six transmembrane helical segments (S1-S6) connected by intra- and extracellular loops (Fig. 1). The S1-S4 helices form the voltage-sensor domain (VSD), while S5-S6 helices line the pore domain (PD). During membrane depolarization, the S4 helix of the VSD slides outwards in the membrane due to its positively charged amino acids, which eventually results in the opening of the activation gate at the intracellular entrance of the PD (19, 20, 21). Prolonged depolarization subsequently leads to the closure of the inactivation gate at the extracellular vestibule of the pore establishing the non-conductive, inactivated state of the channel (18, 20, 22, 23).

**Fig. 1:**
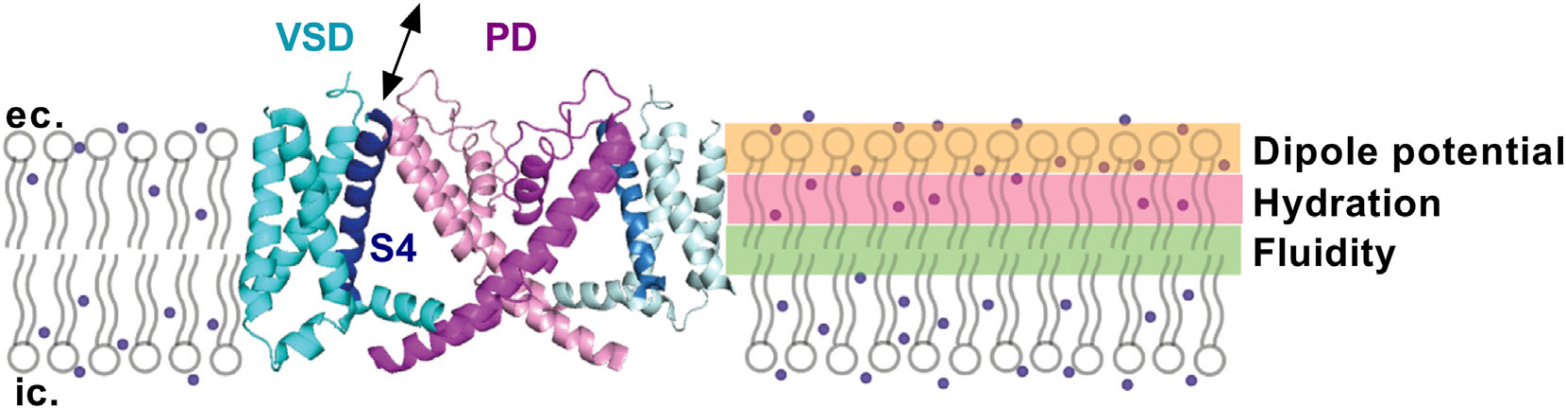
The structure of K_V_1.3 and the major parameters characterizing the biophysical properties of membranes at different depths. S1-S6 helices of two opposing subunits of the human K_V_1.3 ion channel based on the cryo-EM structure are displayed from a lateral view (PDB: 7EJ1 (17), created in PyMol). S1-S4 helices forming the voltage-sensor domain (VSD) are shown in blue colors, while S5-S6 helices forming the pore domain (PD) are in purple. S4 helices of the VSD containing positively charged residues are highlighted in dark blue. In response to the accumulation of positive charges on the intracellular side of the membrane during depolarization, S4 helices move outwards in the membrane (black arrow), which is transmitted to the activation gate formed by the bundle crossing at the intracellular entry of the PD leading to pore opening. These events occur through the permission and cooperativity of the surrounding membrane bilayer. Therefore, molecular organization mainly determined by the lipid composition can influence these transitions in an indirect manner. Three major order-related biophysical parameters, fluidity (green shading), hydration (pink shading) and dipole potential (orange shading) characterize this structural arrangement at different depths of the membrane. Small dots represent water molecules penetrating the membrane to variable depth in the figure. The membrane embedding of the ion channel was sketched based on the K_V_1.2-K_V_2.1 electron microscopic structure in lipid nanodisc (PDB: 6EBK (18)).

In general, inhibitory ceramide effects were proposed previously for various members of the K_V_ channel superfamily, such as K_V_1.5 (24), K_V_2.1 (24, 25), and human-ether-a-go-go-related channel (HERG, K_V_11.1) (26, 27, 28, 29). However, these studies were typically limited to describing the effects on basic properties of the channels (i.e. current amplitude and steady-state activation) without examining the detailed molecular mechanisms. Furthermore, the effects of glucosylceramides on ion channels are practically completely unknown. Membrane ceramides were reported to have inhibitory effects on K_V_1.3 as well by (i) inhibiting whole-cell currents through (ii) decreasing the open probability of the channel without affecting its unitary conductance (30, 31) and (iii) resulting in a positive shift in steady-state activation, thus channel opening occurring at more positive membrane potentials (32). Despite these significant functional changes, it is not known through which molecular structural element of the ion channel these electrophysiological effects of ceramides are transmitted. Furthermore, the degree of contribution of indirect effects is also not elucidated, which would be required for a better understanding of the mechanism of ceramide effects on K_V_ channel gating.

Two-electrode voltage-clamp fluorometry (TEVCF), a unique combination of electrophysiological and fluorescence techniques, can simultaneously track movements of the VSD during the entire gating process after labeling a cysteine residue introduced in the extracellular S3-S4 loop of the VSD with an MTS-conjugated fluorophore, and that of the PD by recording ionic currents (33). Therefore, TEVCF offers an exceptional opportunity to elucidate the complex mechanism of action of lipids by determining the primary intramolecular target: do they target the VSD or the PD to exert their complex electrophysiological effects (2, 9). In our previous study, we employed TEVCF to identify the domain through which the well-known inhibitory effects of membrane sterols are translated into K_V_ channels. We showed that sterol actions are mediated by targeting the PD instead of modifying the VSD of K_V_1.3 and K_V_10.1 (34). According to the proposed model, sterols can inhibit directly the expansion of the pore during activation, which was supported by the slowed current activation kinetics, right-shifted steady-state activation (G-V) curves, reduced current amplitudes, and decreased single channel unitary conductance. Furthermore, these effects were accompanied by no changes in single-channel open probabilities, or parameters characterizing VSD movements including steady-state activation (F-V) curves and activation kinetics of the fluorescence signal. No such studies have been performed for membrane ceramides yet.

While TEVCF provides invaluable information about the intramolecular site of action of lipids, fluorescence-based techniques, and environment-sensitive fluorophores can provide additional insights into the molecular mechanism of the effects by demonstrating the potential presence of alterations in membrane biophysical properties, which can contribute to the modulatory actions of ceramides and glucosylceramides in an indirect manner (2, 35, 36, 37). The biophysical properties of membranes are strongly linked to the molecular arrangement of membrane lipids, which is most commonly characterized by three order-related parameters: membrane fluidity, hydration, and dipole potential that describe structural organization at different depths of bilayers (2, 38, 39, 40) (Fig. 1). These properties essentially depend on the quantity and quality of membrane lipids. For example, an increased abundance of both ceramides and glucosylceramides was previously suggested to reduce fluidity and hydration in living cells (41, 42, 43, 44). Considering the link between membrane biophysics and protein function (1, 2), this raises the possibility that ceramides and glucosylceramides may affect channel function by modulating structural transitions via indirect mechanisms given that molecular rearrangements during channel gating occur at these membrane regions (Fig. 1). Consistently, indirect membrane-related ceramide effects were proposed to contribute to the modulation of Piezo1 (45).

Here, we report on inhibitory actions induced by exogenously loaded membrane-incorporating C16-ceramide (Cer) and C16-glucosylceramide (GlcCer) on the activation gating of the K_V_1.3 ion channel expressed in *Xenopus laevis* oocytes. Using TEVCF, we demonstrate that while both Cer and GlcCer cause rightward shifts in the steady-state activation of the channel (G-V curves), slow down current activation, and decrease current amplitudes, these two lipids exert these effects through distinct molecular mechanisms revealed by the parallel recording of fluorescence signals tracking VSD movements and currents monitoring the PD. We propose that Cer targets the VSD, which is demonstrated by a parallel rightward shift in the steady-state activation of the fluorescent signal (F-V curves) and decelerated fluorescent signal activation kinetics, while GlcCer induces no such changes and, therefore, primarily acts on the PD. Based on our experiments investigating the complex membrane biophysical effects of the two lipids at three different depth layers of the membrane by quantifying effects on fluidity, hydration, and dipole potential, we demonstrate that while both lipids similarly alter membrane organization in the inner layers by decreasing membrane fluidity and hydration, Cer induces dramatic changes at the membrane-water interface manifested in a reduced dipole potential. The latter may paradoxically refer to a strongly ordered local microenvironment that could act against the rearrangement of the VSD at this interfacial region during activation gating.

## Materials and Methods

### Molecular biology, expression systems, and cell lines

The A309C mutant of the human K_V_1.3 (*KCNA3*, Uniprot B2RA23) with a cysteine introduced into the extracellular S3–S4 linker of the channel optimal for TEVCF (34) was constructed through site-directed mutagenesis (QuikChange; Agilent) in a pBSTA vector, and the construct was subsequently verified by sequence analysis. For injection, plasmids coding for the mutant channel were linearized with *Hind*III and transcribed to RNA using the Invitrogen mMESSAGE mMACHINE T7 Transcription Kit (Thermo Fisher Scientific).

For TEVCF experiments, *Xenopus laevis* oocytes obtained from EcoCyte Bioscience (Dortmund, Germany) were injected with 30–50 nl of ∼1 μg/μl RNA and subsequently incubated at 18°C for 1–3 days in ND93 that contained 93 mM NaCl, 5 mM KCl, 1.8 mM CaCl_2_, 1 mM MgCl_2_, 5 mM HEPES and 50 mg/l Gentamycin, with the pH of the solution set to 7.4. All chemicals used for the preparation of the solutions were purchased from Sigma-Aldrich.

For experiments determining potential alterations in membrane biophysical parameters in response to ceramide and glucosylceramide treatments, the Chinese hamster ovary (CHO) cell line was obtained from the American Type Culture Collection (Manassas, VA) and grown according to its specifications. For spectrofluorometry measurements, cells were harvested at a confluence of 80%–90% by trypsinization, while for confocal microscopy, cells were grown on 8-well chambered coverglass (ibidi).

### Cell membrane ceramide and glucosylceramide modulations

For ceramide and glucosylceramide treatments C16-ceramide (Cer, d18:1/16:0, N-palmitoyl-D-erythro-sphingosine) and C16-glucosylceramide (GlcCer, d18:1/16:0, D-glucosyl-β-1,1′-N-palmitoyl-D-erythro-sphingosine) were obtained from Avanti Polar Lipids (Alabaster, AL). Cer and GlcCer loadings were performed at concentrations of 40 μM (the stock dissolved in ethanol with a final solvent concentration less than 0.5% during treatments) diluted in ND93 (for electrophysiology) or cell-growth medium (for spectrofluorometry and microscopy) for 24 h at 18°C for oocytes or 37°C for CHO cells, respectively. The applied concentrations and treatment durations are in accordance with those used in a variety of recent studies (46, 47, 48).

### Two-electrode voltage-clamp fluorometry (TEVCF)

For TEVCF experiments oocytes were labeled for 30 min on ice with 10 μM of 2-((5(6)-tetramethylrhodamine)carboxylamino)ethyl methanethiosulfonate (TAMRA-MTS, Toronto Research Chemicals, Toronto, ON, Canada). The dye was diluted in a depolarizing solution containing 110 mM KCl, 1.5 mM MgCl_2_, 0.8 mM CaCl_2_, 0.2 mM EDTA, 10 mM HEPES, at pH 7.1. After labeling followed by an extensive washing with ND93, the oocytes were stored in the dark on ice before the measurements. For recording, ND93 was applied as the extracellular recording solution in the bath, while a 3 M KCl solution was used as the intracellular solution in the pipettes.

TEVCF recordings were performed using an Oocyte Clamp OC-725C amplifier (Warner Instruments), while fluorescence signals were acquired through a 40×, 0.8-NA CFI Plan Fluor Nikon water-immersion objective on a Nikon Eclipse FNI microscope (Nikon, Tokyo, Japan) and a photodiode (PIN-040A; United Detector Technology, OSI Optoelectronics). TAMRA-MTS signals were measured by using a green (530 nm) M530L2-C1 LED from Thorlabs, a 545/25 excitation filer, a 565LP dichroic mirror, and a 605/70 emission filter. The signal from the photodiode was collected by an Axopatch 200A amplifier and a Digidata-1550 digitizer controlled by pClamp10 (Molecular Devices). The acquired fluorescence traces represent single recordings without averaging and were filtered with a Gaussian filter.

### Analysis of TEVCF data

During TEVCF measurements on K_V_1.3 309C, current and fluorescence signals were recorded at 5 kHz and low-pass filtered at 1 kHz. Fluorescence signals were calculated as ΔF/F in percentage, where ΔF is the change in the signal amplitude, and F is the baseline fluorescence level at the time of the signal. To eliminate distorting effects caused by photobleaching, recorded traces were corrected by subtracting the baseline fluorescence trace exhibiting no voltage-dependent changes. Subsequently, to obtain F_norm_-V curves, fluorescence values were determined from the steady-state components, normalized to the maximum obtained intensity and plotted as a function of test potential.

To generate I-V curves, peak currents were determined, corrected for the leak current and plotted as a function of the applied test potential. Subsequently, V_1/2_ and k parameters of the conductance-voltage (G-V) curves were determined by fitting the equation

$$I=\frac{V\times G_{\max}\times \left(1-e^{-\frac{V-E_{rev}}{25}}\right)}{\left(1-e^{-\frac{V}{25}}\right)\times \frac{1}{1+e^{-\frac{V-V_{1/2}}{k}}}}$$

to I-V curves, which combine Goldman-Hodgkin-Katz rectification with Boltzmann voltage dependence. In the formula, V and I represent the voltage and the current values, respectively, while the G_max_, the maximum conductance, E_rev_ the reversal potential, V_1/2_ and k the half-activation voltage and slope factor of the Boltzmann function, respectively, are the free determined parameters. In each cell, G_norm_ values at given test potentials were calculated using the formula:

$$G\left(V\right)=\frac{1}{1+e^{-\frac{V-V_{1/2}}{k}}}$$

When examining the activation kinetics of ionic currents, the following single exponential function was fitted to the rising phase of the traces to obtain current activation time constants (τ_act_):

$$I=I_{0}\times \left(1-e^{-\frac{t}{\tau_{act}}}\right)+C$$

To determine fast (τ_act,f_) and slow (τ_act,s_) activation time constants of fluorescence signals, the following double exponential function was fitted to the measured data:

$$I=I_{0f}\times \left(1-e^{-\frac{t}{\tau_{act,f}}}\right)+I_{0s}\times \left(1-e^{-\frac{t}{\tau_{act,s}}}\right)+C$$

F_norm_-V curves were quantified by fitting a Boltzmann function:

$$y=\frac{1}{1+e^{-\frac{V-V_{1/2}}{k}}}$$

and determining V_1/2_ and k from the fits for individual cells.

To examine effects induced by Cer and GlcCer loading on current amplitudes in TEVCF experiments, peak currents elicited by a +40 mV depolarizing pulse were determined, corrected for the leak current, and then normalized to the daily mean of leak-corrected currents of control oocytes to obtain relative current amplitudes with eliminating day-to-day variability of currents.

Analyses of electrophysiological data were performed using Clampfit (v10; Molecular Devices), SigmaPlot (v10; Systat Software) and Excel (Microsoft) software.

### Examination of membrane fluidity and hydration

To determine changes in membrane fluidity and hydration in response to Cer and GlcCer, fluorescence anisotropy of 4′-(trimethylammonio)-diphenylhexatriene (TMA-DPH) and generalized polarization (GP) of 6-dodecanoyl-N,Ndimethyl-2-naphthylamine (Laurdan) were quantified as described previously (36, 37). TMA-DPH and Laurdan were purchased from Sigma-Aldrich. Trypsinized control CHO cells and those treated with 40 μM Cer or GlcCer for 24 h at 37°C were washed and labeled in Hank’s buffer with 10 μM TMA-DPH or 2 μM Laurdan for 20 min at room temperature. TMA-DPH labeled cells were diluted in Hank’s buffer without washing to a concentration of 10^6^/ml, while Laurdan-stained cells were washed once and resuspended at a concentration of 10^6^/ml in Hank’s buffer. Fluorescence intensities were measured with a Fluorolog-3 spectrofluorometer (Horiba Jobin Yvon) with the temperature of the cuvette holder adjusted to 37°C by a circulating water bath.

The fluorescence anisotropy (r) of TMA-DPH was determined in the L-format after an excitation at 352 nm and measurement of fluorescence intensities at 430 nm according to the following formula:

$$r=\frac{I_{vv}-{GI}_{vh}}{I_{vv}+{2GI}_{vh}}$$

where I_vv_ and I_vh_ are the vertical and horizontal components, respectively, of the fluorescence excited by vertically polarized light, and G is an instrument-specific correction factor characterizing the different sensitivity of the detection system for vertically and horizontally polarized light:

$$G=\frac{I_{hv}}{I_{hh}}$$

where I_hv_ and I_hh_ are the vertical and horizontal components, respectively, of the fluorescence excited by horizontally polarized light.

Laurdan was excited at 350 nm and its emission was detected in the blue range of its emission spectrum at 435 nm (I_blue_) and at the red edge at 500 nm (I_red_). Generalized polarization (GP) of Laurdan fluorescence was calculated according to the following formula:

$$GP=\frac{I_{blue}-I_{red}}{I_{blue}+I_{red}}$$

### Determination of the membrane dipole potential

To quantify alterations in the magnitude of the membrane dipole potential, an excitation ratiometric assay based on the di-8-ANEPPS (4-(2-[6-(dioctylamino)-2-naphthalenyl]ethenyl)-1-(3-sulfopropyl) pyridinium inner salt) voltage-sensitive fluorophore and confocal microscopy was applied as described previously (35, 49). The di-8-ANEPPS fluorophore was obtained from Thermo Fisher Scientific. In these confocal microscopic measurements, control CHO cells grown on 8-well chambered coverglass and those treated with 40 μM Cer or GlcCer for 24 h at 37°C were washed and labeled with 2 μM di-8-ANEPPS for 20 min at room temperature. After washing, images were taken at the midplane of cells using an LSM880 confocal laser-scanning microscope (Carl Zeiss AG). Di-8-ANEPPS was excited at 458 nm and 514 nm, and its emitted intensities were measured between 584 and 686 nm (I_blue_ and I_red_ after excitation at 458 nm and 514 nm, respectively). During quantitative image analysis, images were segmented into membrane and nonmembrane pixels with the manually seeded watershed algorithm using a custom-written MATLAB program. The average value of the fluorescence intensity ratio (*R*_*exc*_ *= I*_*blue*_*/I*_*red*_) positively correlating with the magnitude of dipole potential was calculated from the data of cell membrane pixels after background subtraction for each image containing data of 30–40 cells of normal morphology.

### Statistical analysis

The reported errors are SEM, and the numbers of independent samples (n) involved in the given analyses are shown in the text. All electrophysiological experiments were carried out on n oocytes originating from at least 4 different frogs. Spectro fluorometry and confocal microscopy data were obtained in three independent experiments. In spectrofluorometry, each of the n samples contained approximately 100,000 cells. In confocal microscopy analysis, for each treatment n number of images from five independent experiments were recorded each containing 30–40 cells per image. The *P* values were calculated by Tukey's HSD test carried out after significant differences were obtained for between-group effects in ANOVA. Differences were considered significant (asterisk, ∗) when *P* < 0.05.

## Results

### Effects of Cer and GlcCer on voltage-dependent steady-state activation of the pore domain and the voltage sensor of K_V_1.3

**Fig. 2:**
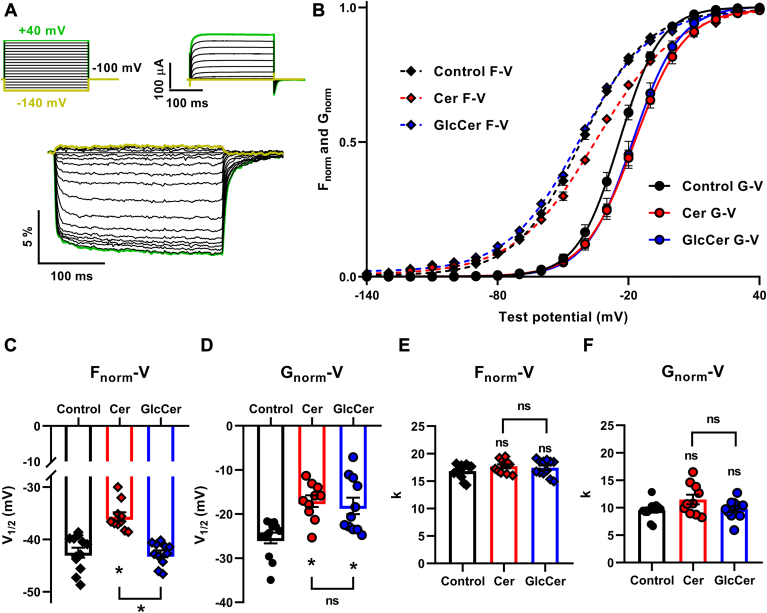
Effects of Cer and GlcCer on voltage-dependent steady-state gating parameters of K_V_1.3. A: to determine steady-state activation of fluorescent signals and ionic currents, A309C channels were expressed in *Xenopus laevis* oocytes and labeled with MTS-TAMRA. Control oocytes and those loaded with 40 μM Cer or GlcCer for 24 h were depolarized from a holding potential of −100 mV to 250-ms long test potentials ranging from −140 to +40 mV in steps of 10 mV every 30 s. Voltage protocols (top left panel), evoked representative current traces (top right panel) and fluorescence signals (bottom panel) are shown. B: mean ± SEM of the normalized fluorescence change (F_norm_) and normalized conductance (G_norm_) values were calculated in n = 10–12 cells and plotted as a function of the applied test potential (F_norm_-V and G_norm_-V curves, respectively). The superimposed dashed (F_norm_-V) and solid (G_norm_-V) lines show best fitted representative Boltzmann functions to the data points. C*–*F: half-activation voltage (V_1/2_) and slope factor (k) values determined from both F_norm_-V and G_norm_-V curves in n = 10–12 individual cells, and their average values (±SEM) are plotted. Asterisks (∗) indicate significant differences compared to control samples, or between samples treated with Cer and GlcCer (*P* < 0.05, ANOVA followed by Tukey’s HSD test).

For TEVCF measurements, we employed the same experimental setup that we previously applied for examining the mechanism of cholesterol actions by expressing the A309C mutant K_V_1.3 channel with a cysteine introduced in the extracellular S3-S4 linker close to the top of S4 helix of the channel in *Xenopus laevis* oocytes. This mutant exhibits the basic gating properties of the wild-type channel. The introduced cysteine residues were labeled with MTS-TAMRA to study the movements of the voltage sensor, which induces no changes in the electrophysiological parameters of K_V_1.3 (34). Using TEVCF we simultaneously recorded ionic currents to obtain current-voltage (I-V) and subsequently, steady-state activation-voltage (G-V) curves, and fluorescence signals to determine relative fluorescence change-voltage (ΔF/F-V) curves in the −140 to +40 mV range in 10 mV steps. In each step, oocytes were depolarized to the given test potential for 250 ms every 30 s from a holding potential of −100 mV (Fig. 2A top left panel). Representative current and fluorescent signal traces are displayed in Fig. 2A top right and bottom panels, respectively. In Fig. 2B the mean values ± SEM of F_norm_ and G_norm_ are displayed as the function of test potentials with a representative Boltzmann fit (Fig. 2B). Midpoints (V_1/2_) and slope factors (k) of F_norm_-V and G_norm_-V curves were determined from data of individual cells (Fig. 2C–F). In keeping with VSD activation occurring at more negative potentials than pore opening, the F_norm_-V curves appear at more negative potentials compared to the G_norm_-V curves even in control conditions (for example in control cells midpoints were V_1/2_ = −42.5 ± 1.0 mV, n = 12 for F_norm_-V and V_1/2_ = −25.4 ± 1.2 mV, n = 12 for G_norm_-V curves).

In addition to control cells, we also determined these parameters in cells loaded with 40 μM Cer or GlcCer for 24 h. When compared to controls, Cer induced a rightward shift in the F-V curve characterizing movements of the VSD, while GlcCer exerted no such effects (Fig. 2B). Changes in F-V curves can be quantified by determining the midpoints of the curve, i.e. the voltages at which half-maximal changes in the fluorescence signal occur (V_1/2_). In comparison with control cells (V_1/2_ = −42.5 ± 1.0 mV, n = 12), the midpoint determined in Cer-loaded cells was significantly less negative (V_1/2_ = −35.7 ± 0.9 mV, n = 10, *P* < 0.0001), while that of GlcCer-loaded oocytes was not statistically different (V_1/2_ = −42.7 ± 0.7 mV, n = 11, *P* = 0.9824) (Fig. 2C). On the contrary, both Cer and GlcCer induced rightward shifts in G-V curves characterizing pore opening (Fig. 2B), which were also demonstrated by significant changes in the midpoints of the curves since the voltage of half-maximal activation of control cells (V_1/2_ = −25.4 ± 1.2 mV, n = 12) was more negative than that of cells treated with Cer (V_1/2_ = −17.1 ± 1.3 mV, n = 10, *P* = 0.0013) or GlcCer (V_1/2_ = −18.2 ± 1.9 mV, n = 11, *P* < 0.0038) (Fig. 2D). No changes were observed in k values characterizing the slopes of either F-V (k = 16.8 ± 0.4, n = 12 for control vs. k = 17.7 ± 0.4, n = 10, *P* = 0.2658 for Cer; and k = 17.4 ± 0.4, n = 11, *P* = 0.5167 for GlcCer) or G-V curves (k = 9.6 ± 0.5, n = 12 for control vs. k = 11.5 ± 0.9, n = 10, *P* = 0.0907 for Cer; and k = 9.8 ± 0.5, n=11, *P* = 0.9698 for GlcCer) (Fig. 2E, F). These results imply that while the two lipids have the same effect on the PD and shift channel activation threshold to more positive membrane potentials represented by rightward shifts in steady-state activation (G-V) curves, Cer has the same effect on the steady-state activation of the VSD (F-V curves) while GlcCer affects primarily the pore.

### Effects of Cer and GlcCer on current and VSD activation kinetics of K_V_1.3

**Fig. 3:**
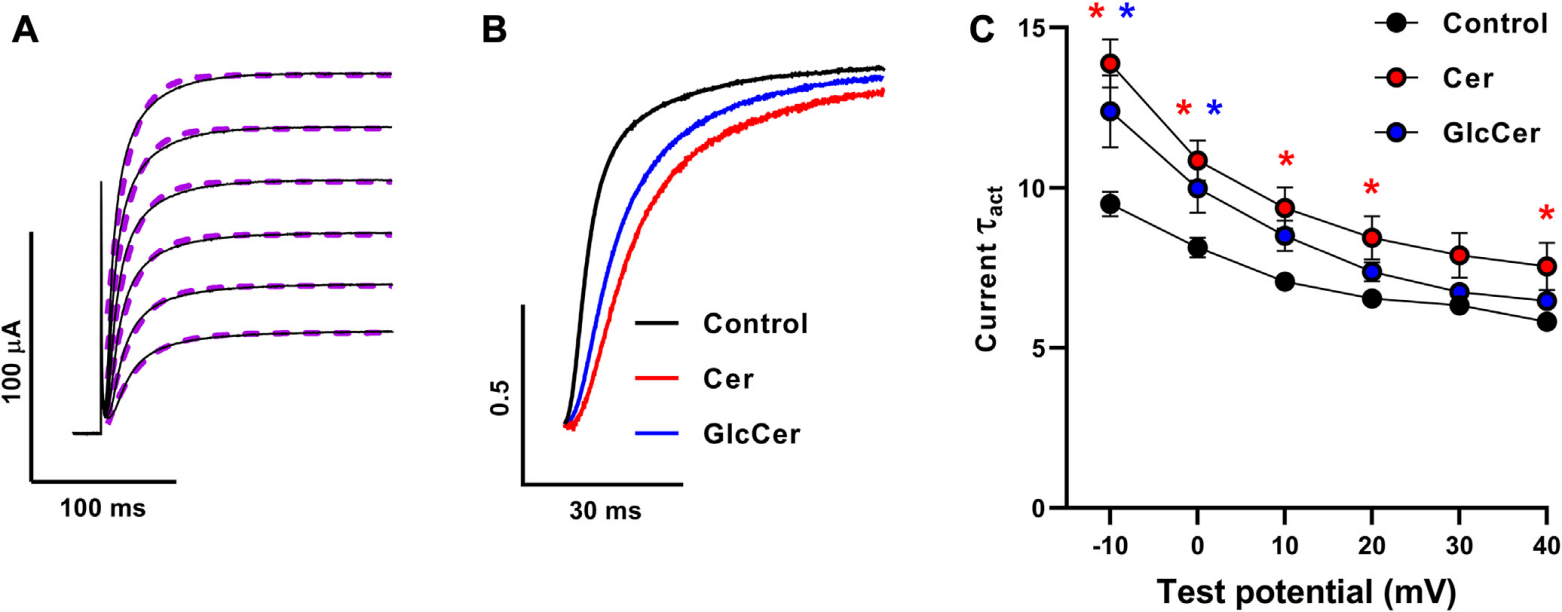
Effects of Cer and GlcCer loading on K_V_1.3 current activation kinetics. A: current traces evoked by 250-ms depolarizing test potentials in the −10 to +40 mV range in 10 mV steps every 30 s from a holding potential of −100 mV obtained in *Xenopus laevis* oocytes were well fitted by single exponential functions. B: representative normalized current traces elicited by −10 mV depolarization in a control oocyte, and those treated by 40 μM Cer or GlcCer for 24 h are shown. C: activation time constants (τ_act_) of ionic currents evoked by test potentials between −10 and +40 mV were determined by fitting single exponentials in n = 10–12 individual cells, and their average values (±SEM) are plotted. Asterisks (∗) indicate significant differences compared to control samples at the given test potential (*P* < 0.05, ANOVA followed by Tukey’s HSD test).

Next, we examined the effects of Cer and GlcCer on VSD activation and channel opening kinetics by applying the same pulse protocol as shown in Fig. 2A. When examining current activation mirroring pore opening, we analyzed traces evoked by test potentials between −10 and +40 mV, which were well fitted by single exponential functions (Fig. 3A). Both Cer and GlcCer treatment resulted in slowed current activation kinetics as illustrated by the representative normalized current traces in response to −10 mV test potentials, in which the activation was slower in both Cer- and GlcCer-treated oocytes compared to controls (Fig. 3B). A detailed statistical analysis of activation time constants (τ_act_) of the evoked ionic currents in individual cells showed that Cer resulted in significantly higher current τ_act_ values than that observed in controls at almost all applied voltages, whereas the GlcCer-induced elongation was typically at the limit of statistical significance with significant changes observed at −10 and 0 mV test potentials (Fig. 3C).

**Fig. 4:**
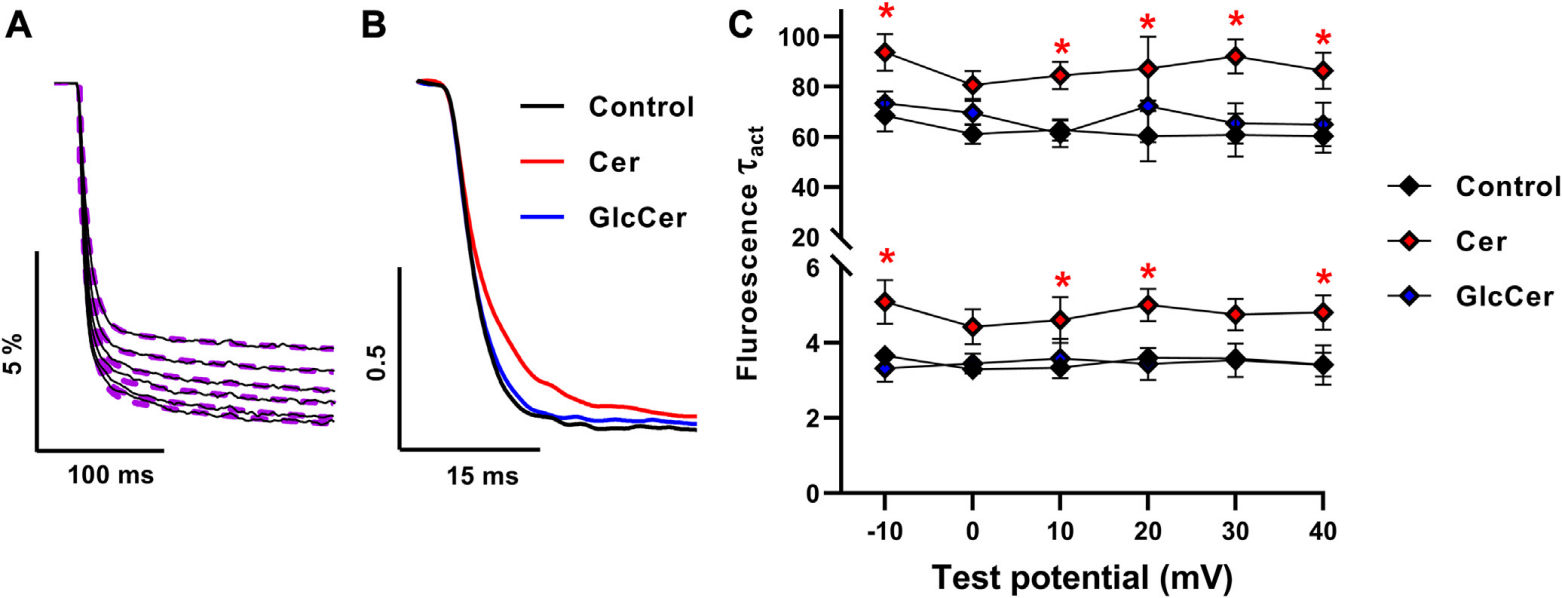
Effects of Cer and GlcCer on K_V_1.3 VSD activation kinetics. A: fluorescence signal traces evoked by 250-ms depolarizing test potentials in the −10 to +40 mV range in 10 mV steps every 30 s from a holding potential of −100 mV obtained in *Xenopus laevis* oocytes were well fitted by double exponential functions. B: representative normalized fluorescent signal traces elicited by −10 mV test potentials in control oocytes, and those loaded by 40 μM Cer or GlcCer for 24 h are shown. C: activation time constants (τ_act_) of fluorescent signals evoked by test potentials between −10 and +40 mV and fitted by double exponentials determined in n = 10–12 individual cells, and their average values (±SEM) are plotted. Asterisks (∗) indicate significant differences compared to control samples at the given test potential (*P* < 0.05, ANOVA followed by Tukey’s HSD test).

Concomitantly, we also analyzed the kinetics of fluorescence signals corresponding to VSD activation induced by test potentials in the range above. In accordance with results obtained in our previous study (34), fluorescence signals had a fast component accounting for the majority (>85%) of the amplitude and a lower-amplitude, slow component. Accordingly, fluorescence signals were well fitted with a double exponential function (Fig. 4A). As opposed to current activation kinetics, only Cer caused slower fluorescence signal activation as illustrated by the representative fluorescence signal traces induced by −10 mV test potentials compared to control oocytes, whereas no such changes were found in traces recorded in GlcCer-treated oocytes (Fig. 4B). Consistently, when analyzing both activation time constants (τ_act_) of double exponential fits quantitatively, Cer resulted in higher time constants characterizing fast and slow components of VSD activation, which were statistically significant in both time constants at almost all examined test potentials. Conversely, GlcCer treatment led to no significant changes in either of the fluorescence signal τ_act_s at any examined test potential (Fig. 4C). The amplitude ratios of the two components did not change in response to either treatment (data not shown).

The fact that Cer decelerated activation kinetics of both VSD activation and pore opening, while GlcCer modulated only the latter, corroborates our hypothesis raised based on our results obtained when examining voltage-dependent steady-state parameters (G-V and F-V curves) of K_V_1.3, that is, Cer is able to directly target the VSD, whereas GlcCer affects the PD without modifying the function of the VSD.

### Effects of Cer and GlcCer on K_V_1.3 current amplitudes

**Fig. 5:**
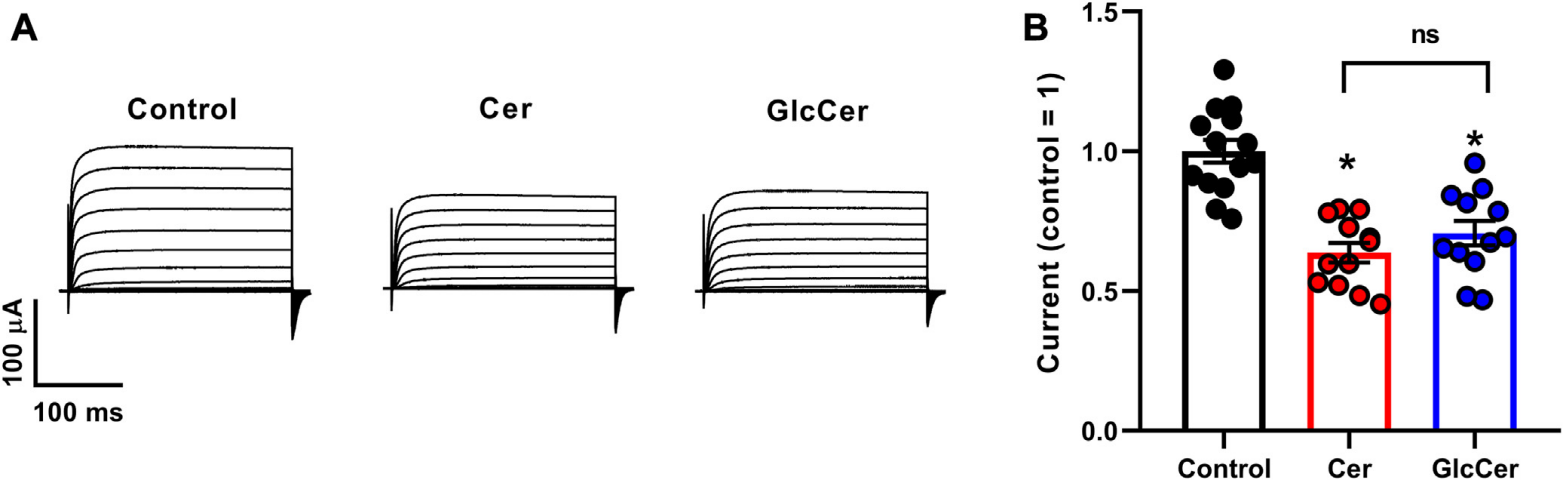
Effects of Cer and GlcCer on K_V_1.3 current amplitudes. A: representative current traces evoked by 250-ms depolarizing test potentials in the −140 to +40 mV range in 10 mV steps every 30 s from a holding potential of −100 mV obtained in a control *Xenopus laevis* oocyte and those treated by 40 μM Cer or GlcCer for 24 h on the same day. B: amplitudes of currents evoked by depolarizing pulses to +40 mV in n = 12–14 control and Cer- or GlcCer-treated oocytes were normalized to the daily mean of control cells and the values of individual cells, and their average values (±SEM) were plotted. Asterisks (∗) indicate significant differences compared to control samples, or between samples treated with Cer and GlcCer (*P* < 0.05, ANOVA followed by Tukey’s HSD test).

We also examined the effects of Cer and GlcCer loading on the amplitude of K_V_1.3 currents in the *Xenopus laevis* expression system. Although we did not determine current densities due to the lack of cell capacitance information, we measured currents after injection of identical amounts of K_V_1.3 mRNA and recorded on the same day, which were generally decreased by ∼30–40% in the treated cells at all examined voltages (Fig. 5A). When comparing current amplitudes elicited by the maximal +40 mV pulses and normalized to the daily mean of control cells (1.000 ± 0.041, n = 14), the values were significantly reduced both in response to Cer (0.637 ± 0.036, n = 12, *P* < 0.0001) and GlcCer (0.707 ± 0.044, n = 12, *P* < 0.0001) (Fig. 5B). Current amplitudes did not differ significantly between oocytes treated with Cer and GlcCer (*P* = 0.4658).

### Effects of Cer and GlcCer on membrane fluidity and hydration

**Fig. 6:**
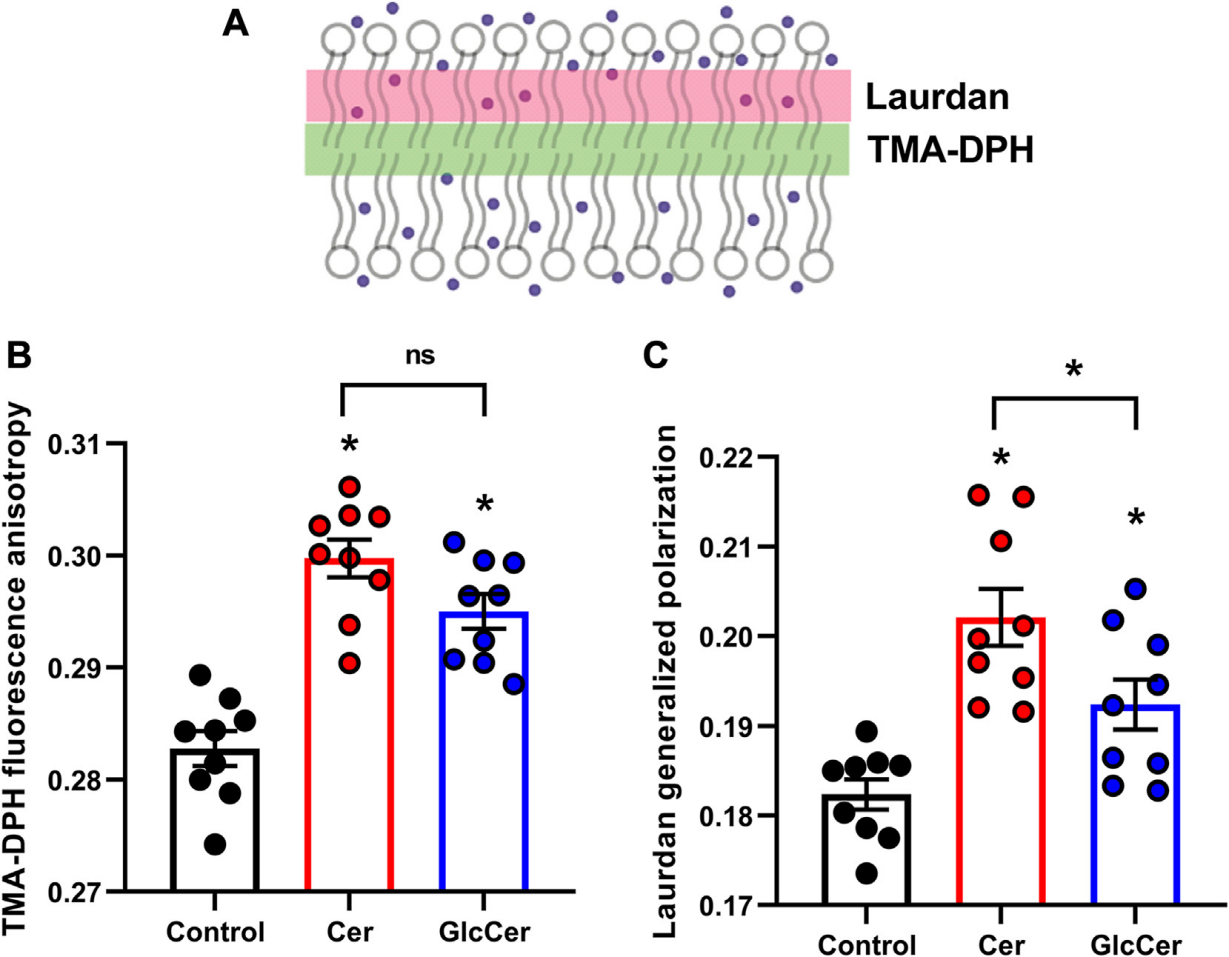
Effects of Cer and GlcCer on membrane fluidity and hydration. A: membrane fluidity and hydration are two order-related parameters characterizing the molecular organization in the central hydrophobic core of the membrane and in regions between the core and the membrane-water interface, respectively. While membrane fluidity can be examined by quantifying the fluorescence anisotropy of TMA-DPH, hydration is measured by determining the generalized polarization of Laurdan. B: control CHO cells and those treated with 40 μM Cer or GlcCer for 24 h were labeled with TMA-DPH and the fluorescence anisotropy of the fluorophore was determined using spectrofluorometry. C: alternatively, control cells and those treated as above were labeled with Laurdan, followed by quantification of generalized polarization (GP) of the dye with spectrofluorometry. TMA-DPH anisotropy and Laurdan GP values were obtained in n = 9 independent samples containing approximately 100,000 cells, and their average values (±SEM) are plotted in panels B and C, respectively. Asterisks (∗) indicate significant differences compared to control samples, or between samples treated with Cer and GlcCer (*P* < 0.05, ANOVA followed by Tukey’s HSD test).

Results of the previous experiments show that, in general, Cer and GlcCer exert inhibitory actions on the K_V_1.3 ion channel, however, they may target different domains of the channel and thus may act through distinct mechanisms. While TEVCF measurements identified the main intramolecular target of the two lipids, that is, the VSD for Cer and the PD for GlcCer, these experiments did not provide information about the potential contribution of indirect lipid effects. Thus, an investigation of membrane biophysical parameters is warranted, which could further elucidate their mechanism of action. Although Cer and GlcCer have previously been described to affect the biophysical properties of lipid bilayers, a thorough analysis of such changes in different layers in which the molecular rearrangements of the ion channels during gating take place has not been performed. Therefore, we carried out experiments to examine alterations in membrane biophysical parameters occurring at various bilayer depths by manipulating Cer and GlcCer levels of the cell membrane. In these measurements we applied CHO cells given that *Xenopus laevis* oocytes are not suitable for well-reproducible fluorescence-based membrane biophysical measurements due to their size, vitelline membrane and their pigmented animal and non-pigmented vegetal poles. First, we investigated two order-related parameters, membrane fluidity and hydration that describe molecular organization in the interior hydrophobic core (Fig. 6A, green rectangle) and in layers between the core and the membrane-water interface (Fig. 6A, pink rectangle), respectively.

When examining membrane fluidity, control CHO cells and those loaded with 40 μM Cer or GlcCer were labeled with TMA-DPH and the fluorescence anisotropy of the fluorophore was quantified using spectrofluorometry (36, 37). When compared to TMA-DPH anisotropy of control cells (0.283 ± 0.002, n = 9) the values obtained after Cer and GlcCer treatments were significantly higher (Cer: 0.300 ± 0.002, n = 9, *P* < 0.0001; and GlcCer: 0.295 ± 0.002, n = 9, *P* < 0.0001) implicating reduced fluidity (Fig. 6B). TMA-DPH anisotropy of samples treated with Cer and GlcCer did not significantly differ from each other (*P* = 0.1092).

The hydration of bilayers was examined by labeling CHO cells with Laurdan and quantifying the generalized polarization (GP) of the dye with a spectrofluorometer (36, 37). Consistent with the results obtained with TMA-DPH, increasing cellular Cer or GlcCer levels resulted in higher Laurdan GP values than that observed in untreated cells (control: 0.182 ± 0.002, n = 9 vs. Cer: 0.202 ± 0.003, n = 9, *P* < 0.0001; and GlcCer: 0.192 ± 0.003, n = 9, *P* = 0.0326) (Fig. 6C). These values mirrored reduced membrane hydration after Cer and GlcCer treatments. Furthermore, cells treated with Cer showed higher Laurdan GP, i.e. lower hydration, compared to that after GlcCer (*P* = 0.0388).

In conclusion, Cer and GlcCer similarly modified the interior layers of the cell membrane, as mirrored by reduced membrane fluidity and hydration.

### Effects of Cer and GlcCer on membrane dipole potential

**Fig. 7:**
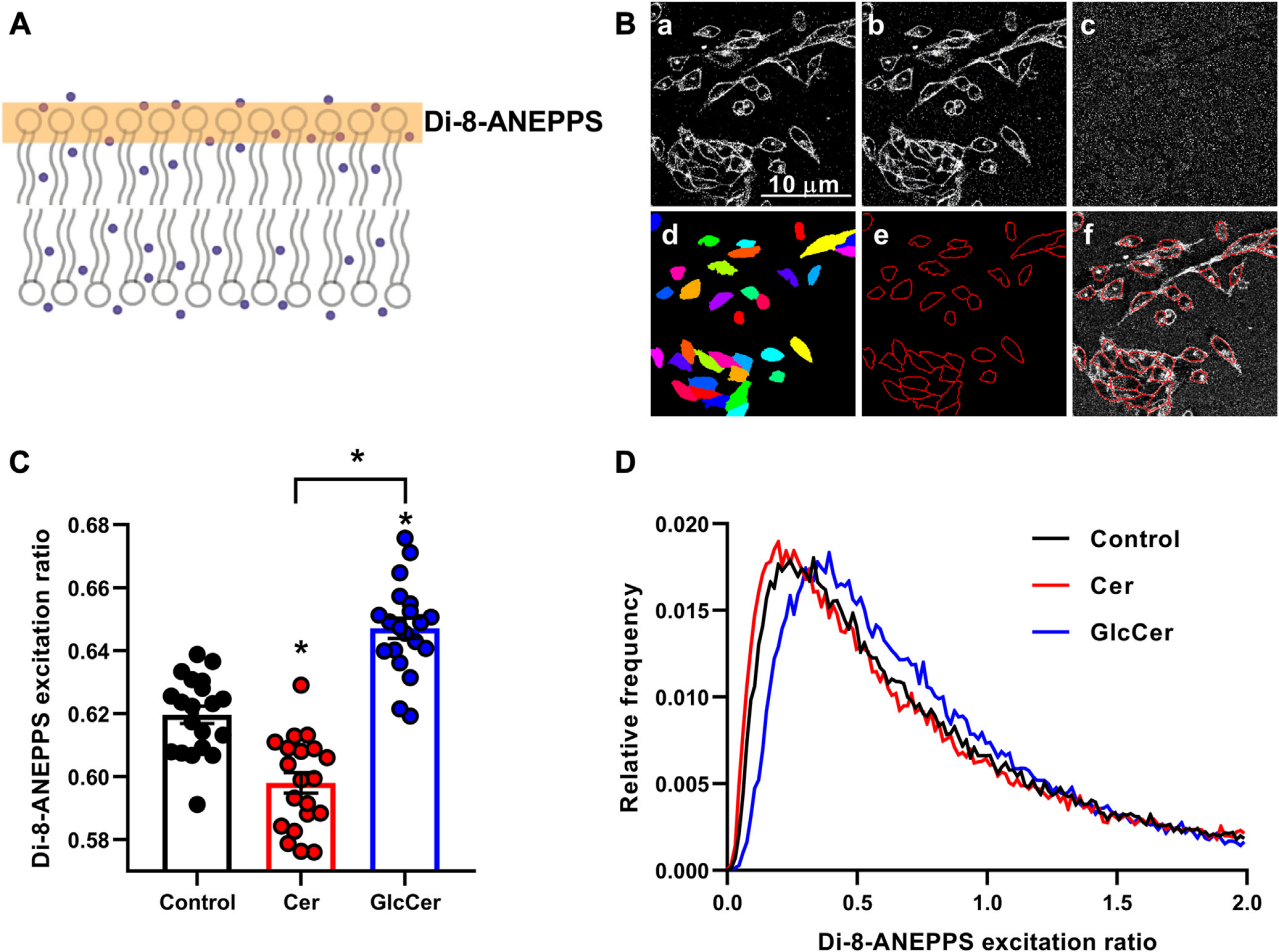
Effects of Cer and GlcCer on membrane dipole potential. A: membrane dipole potential arising from the preferential arrangement of molecular dipoles at the membrane-water interface can be quantified by determining the excitation ratio of the di-8-ANEPPS voltage-sensitive fluorophore using confocal microscopy. B: control CHO cells and those treated with 40 μM Cer or GlcCer for 24 h were labeled with di-8-ANEPPS. Representative confocal microscopic images taken at the midplane of cells show di-8-ANEPPS intensities measured after excitation at two different wavelengths (a, b) and the excitation ratio calculated on a pixel-by-pixel basis (c). During quantitative image analysis a custom-written manually seeded watershed algorithm identified the individual cells (d) and consequently pixels corresponding to the cell membrane (e). The representative image shows the overlay of the membrane mask and the recorded image (f). C: mean excitation ratio values of 20 individual images obtained in five independent experiments and their average values (±SEM) are plotted for the different treatments. Each image contained data of 30–40 cells of normal morphology with a total number of 600–800 cells per treatment. Asterisks (∗) indicate significant differences compared to control samples, or those between samples treated with Cer and GlcCer (*P* < 0.05, ANOVA followed by Tukey’s HSD test). D: representative histograms demonstrate pixelwise distributions of the di-8-ANEPPS excitation ratio in control samples and those treated with Cer and GlcCer.

In the next step of our experiments, we examined the effects of Cer and GlcCer treatments on the magnitude of dipole potential which is another order-related parameter of biological bilayers, however, it reports on the molecular organization more peripherally at the membrane-water interface (Fig. 7A, orange rectangle). In these measurements, we labeled control and Cer- or GlcCer-treated CHO cells with the di-8-ANEPPS fluorophore and quantified the excitation ratio of the dye using confocal microscopy, which positively correlates with the magnitude of the dipole potential (35, 49, 50). During quantitative image analysis, we identified plasma membrane pixels of each cell and determined the di-8-ANEPPS excitation ratio from data of these pixels only (Fig. 7B) so that this method, unlike the previously applied TMA-DPH- or Laurdan-based techniques, gave information selectively from the organization of the plasma membrane.

In these measurements, the mean di-8-ANEPPS excitation ratio of the plasma membrane of control cells (0.620 ± 0.003, n = 20 images, each containing data of 30–40 individual cells) was significantly lower than that of cells treated with GlcCer (0.647 ± 0.003, n = 20, *P* < 0.0001) demonstrating that an increase in the plasma membrane GlcCer level elevates the dipole potential (Fig. 7C). On the contrary, Cer accumulation in the plasma membrane somewhat surprisingly resulted in lower di-8-ANEPPS excitation ratios compared to control cells (0.598 ± 0.003, n = 20, *P* < 0.0001), which, unexpectedly, refers to a reduced dipole potential in response to elevated Cer levels. These changes are also visible in representative di-8-ANEPPS excitation ratio histograms demonstrating data of individual pixels as illustrated by a rightward and a leftward shift induced by GlcCer and Cer, respectively, when compared to the control (Fig. 7D). Considering that dipole potential, similarly to fluidity and hydration, is an order-related parameter, all three are expected to change in a congruent manner as observed in the case of GlcCer. Our results obtained with di-8-ANEPPS after Cer loading are surprising and presumably reflect a massive structural reorganization induced by Cer at the membrane-water interface that will be addressed further in the discussion.

## Discussion

Given that the operation of voltage-gated ion channels involves large-scale conformational changes associated with molecular rearrangements of their transmembrane domains, both the VSD and the PD, the plasma membrane can exert remarkable modulatory actions on the gating process via its lipid composition (Fig. 1). Qualitative and quantitative changes in membrane lipids alter membrane biophysical parameters, which can modulate structural rearrangements in the channel via indirect mechanisms (1, 2). For example, altered fluidity and elasticity can change the mechanosensation of various ion channels including Piezo and volume-regulated anion channels (VRACs) (3, 51), a higher dipole potential can modify voltage sensation (52) or hinder pore movements of K_V_ channels (34), while reduced fluidity and increased membrane lateral stress were suggested to favor the closed state of BK channels leading to reduced open probabilities (53, 54). In accordance, sphingomyelinase-induced ceramide production was demonstrated to establish the non-inactivating behavior of Piezo1 in endothelial cells resulting from indirect membrane-related effects, which enables the sustained activity of the channel contributing to blood pressure regulation (45). However, much less is known about the effects of membrane ceramides including C16-ceramide (Cer) and, in particular, glucosylceramides such as C16-glucosylceramide (GlcCer) on the activation gating of K_V_ ion channels. Therefore, the aim of this study was to (i) examine the electrophysiological effects of these lipids on K_V_1.3, a characteristic member of the K_V_ channel superfamily; (ii) identify the structural element of the channel, VSD or PD, through which the electrophysiological effects of these lipids are mediated; and (iii) support or rule out the potential contribution of indirect effects, lipid-induced alterations in membrane biophysical parameters, to the mechanisms of action.

First, we examined Cer- and GlcCer-induced changes in voltage-sensitive steady-state activation and kinetic parameters of pore opening and VSD activation of K_V_1.3 by a simultaneous recording of ionic currents and fluorescence signals representing PD and VSD movements, respectively. We used the TEVCF technique to describe Cer- and GlcCer-induced electrophysiological effects and identify the intramolecular target of modulation (33). We applied a K_V_1.3 construct with an A309C mutation optimal for MTS-TAMRA labeling and TEVCF measurements, which exhibits conserved gating properties similar to that of the wild-type channel (34). Both Cer and GlcCer induced rightward shifts in G-V curves characterizing steady-state activation resulting in a shift of the activation threshold of the channel towards more positive potentials, and also in depolarizing shifts in V_1/2_ values characterizing half activation potentials (Fig. 2). Furthermore, both lipids decelerated current activation kinetics demonstrated by higher τ_act_ time constants and significantly decreased whole-cell currents (Figs. 3 and 5). The magnitude of changes in these parameters was comparable to that observed previously in response to cholesterol in the same experimental setup indicating the relevance of our findings (34). When examining the VSD movements during activation by analyzing the voltage dependence of the fluorescence signal, we found that Cer resulted in a rightward shift of the F-V curves similar to that observed in G-V curves (Fig. 2), and, in addition, increased fluorescence τ_act_ values referring to slower activation kinetics of VSD, while in the case of GlcCer we did not observe such effects (Fig. 4). Altogether, our TEVCF measurements suggest that while both Cer and GlcCer exert inhibitory effects on K_V_1.3 in general, their exact mechanisms of action considerably differ by affecting distinct domains of the channel. Namely, Cer modifies channel activation gating by primarily acting on the VSD, while GlcCer modifies only the PD during channel activation.

Ceramide-induced alterations observed in TEVCF measurements (a rightward shift in the G-V curve, slowed current activation kinetics, and a reduced current amplitude) are consistent with previous reports on ceramide actions on K_V_1.3 (30, 31). Our observation that ceramide directly affects the VSD is also supported by a previous study demonstrating that ceramides can reduce K_V_1.3 single-channel open probability without affecting unitary conductance (31). Direct pore targeting of GlcCer is supported by the fact that it modifies the examined parameters in the same pattern as cholesterol. Previously, using the same K_V_1.3 mutant and experimental setup, we demonstrated that increasing membrane cholesterol levels resulted in reduced whole-cell currents, slowed current activation kinetics, and a significant shift in the G_norm_-V curve without any effects on F_norm_-V curves or fluorescent signal activation kinetics. These indicated that cholesterol exerts its effects on channel activation gating by primarily affecting the PD instead of acting on the VSD (34).

TEVCF measurements demonstrated that Cer acts through primarily the VSD, and GlcCer affects only the PD (Fig. 2, Fig. 3, Fig. 4), which points to a distinctive mechanism of action of these lipids on K_V_1.3 activation gating. To test if different effects induced by Cer and GlcCer may be related to their distinctive effects on membrane biophysical parameters, we examined such changes in response to these lipids in living CHO cells. Lipids generally affect transmembrane proteins such as ion channels by direct ligand-like binding and indirect actions mediated through alterations in the biophysical properties of membranes (1, 2). The latter parameters are intrinsically associated with the molecular arrangement of membrane constituents, thus substantially depend on bilayer lipid composition. Fluidity, hydration, and dipole potential are the most widely studied biophysical properties in cellular studies and although all of them are considered order-related properties, they describe the structural organization at different depths of bilayers (Fig. 1). Although these parameters are intrinsically related to each other and the membrane regions that they characterize presumably overlap, they are considered separately for didactic reasons throughout the manuscript. Membrane fluidity, defined in a narrower sense as motional freedom of membrane constituents, characterizes lipid packing mainly in the central hydrophobic bilayer core, while hydration, is the extent of water penetration into the membrane, rather in layers between the core and interfacial regions. In living cells, the two parameters are most commonly examined by quantifying the anisotropy and generalized polarization of environment-sensitive fluorophores such as TMA-DPH and Laurdan, respectively (39, 40) (Fig. 6A). In spite of its hydrophilic trimethylammonium group, TMA-DPH is localized in the membrane in a way that its anisotropy reports on membrane structure in the hydrophobic core (55), while the fluorescence spectrum of Laurdan is sensitive to the amount of water molecules surrounding its fluorescent moiety, below the glycerol backbone of the phospholipids (56). Packing order mirrored by fluidity and hydration can affect protein function via different mechanisms. For example, ordering can modify the rate of conformational changes, or influence the relative stability of certain protein conformers according to the hydrophobic mismatch and elastic coupling theories through altering thickness, elastic moduli, and spontaneous curvature of bilayers (38, 40). The least-known of the three above-mentioned parameters, the dipole potential, arises due to the non-random orientation of molecular dipoles at the membrane-water interface leading to the generation of a large positive intramembrane potential (Fig. 7A). Interaction of charged or polar residues of membrane proteins with the electric field of the dipole potential can in turn modify the conformational stabilities thereby shifting the equilibrium between protein states of different functional activities (2, 57). In living cells, changes in the magnitude of dipole potential are monitored by voltage-sensitive fluorophores embedded into the membrane in a way that their fluorescence properties are determined by the strength of the local electric field mainly arising from the dipole potential (35, 37, 49, 50, 58). We have examined membrane fluidity, hydration and dipole potential by quantifying fluorescence anisotropy of TMA-DPH, generalized polarization of Laurdan and excitation ratio of di-8-ANEPPS, respectively. To examine changes occurring in the plasma membrane, we applied short staining periods and fast measurement times for experiments carried out with TMA-DPH and Laurdan to avoid dye internalization, while the quantitative image analysis method ensured that the plasma membrane is exclusively investigated with di-8-ANEPPS.

In terms of membrane biophysics, we observed elevated TMA-DPH fluorescence anisotropy (Fig. 6B) and Laurdan GP (Fig. 6C) values in response to treatment with exogenous Cer referring to reduced membrane fluidity and hydration, respectively. These results are in accordance with previous studies demonstrating Cer-induced lipid ordering in model membranes (59, 60), and in living cells as well in response to treatment with bacterial sphingomyelinase, overexpression of ceramide synthase, or exogenous Cer loading (42, 43). GlcCer was previously shown to also induce ordering in model bilayers (44, 61). Consistently, chemically induced GlcCer accumulation in fibroblasts and macrophages resulted in reduced fluidity and hydration, and an elevated dipole potential (35, 41, 44). Here, we observed similar changes in response to loading CHO cells with exogenous GlcCer (Figs. 6 and 7). These membrane biophysical alterations resembled those caused by cholesterol observed previously (36, 37). GlcCer also elicited changes in the electrophysiological properties of K_V_1.3 (Fig. 2, Fig. 3, Fig. 4, Fig. 5), which were remarkably similar to those observed after cholesterol accumulation with the same experimental setup (34). The fact that the electrophysiological effects on K_V_1.3 by GlcCer and cholesterol, two structurally unrelated lipids, were nearly identical suggests that similar alterations in membrane biophysical properties such as fluidity, hydration, and dipole potential, can markedly contribute to these actions underlining the importance of indirect lipid effects. In our experiments, Cer-induced changes were slightly larger in magnitude compared to that caused by GlcCer with their difference reaching the level of statistical significance when examining membrane hydration (Fig. 6C).

Although the abovementioned structural changes induced by Cer and GlcCer in the hydrophobic core regions of bilayers were roughly congruent, their effects on membrane dipole potential were substantially different (Fig. 7). In contrast to GlcCer accumulation, Cer loading resulted in a lower di-8-ANEPPS excitation ratio referring to a reduced dipole potential and thus suggesting substantially distinctive interfacial actions of the two lipids. This observation might be surprising at first given the intrinsic connection between the dipole potential and lipid ordering (2, 57). We propose a theory to reconcile the apparent contradiction, according to which the unique cone-shaped structure of Cer consisting of a small hydrophilic head much thinner than the width of the hydrophobic tails can lead to a special molecular organization at the membrane-water interface. Resulting from this shape, Cer has a strong tendency to occupy the spaces between the lipid acyl chains giving rise to high packing density and favoring negative spontaneous curvature of bilayers (62, 63). Furthermore, due to the extra hydroxyl group compared to diacylglycerol, the headgroups of Cer can create a dense intermolecular network of multiple H-bonds with sphingomyelin headgroups in a way that is not accessible to other lipid classes (64, 65). In addition, MD simulations suggested that no water molecules are tightly bound to Cer molecules and are highly mobile instead, therefore, Cer headgroups have a preference to be dehydrated (62, 66). Consequently, the amount of structurally arranged interfacial water molecules may be very low in the presence of Cer, and, considering that ordered water molecules provide a major contribution to the generation of dipole potential (2, 57), this may eventually result in a reduced dipole potential. Our results obtained with di-8-ANEPPS clearly suggest that the dramatic ordering effects of Cer on the structural organization of the membrane-water interface may be considerably different from those induced by GlcCer or cholesterol.

As a major limitation of our study, we did not examine possible direct ligand-like interactions between K_V_1.3 and Cer or GlcCer. Strongly concordant changes in TEVCF data induced by cholesterol and GlcCer, two structurally distinct lipids causing similar biophysical alterations in the membrane, support the dominant contribution of indirect mechanisms in their action. Besides the above-described membrane biophysical effects, the distinct intramolecular target of Cer may also be explained by direct binding between the VSD of K_V_1.3 and Cer. Such direct ceramide-ion channel interactions were previously proposed by molecular dynamics simulations in Piezo2, VDAC1, and VDAC2 channels (67, 68). Furthermore, molecular dynamics simulations elucidated specific ceramide binding locations with various, mainly hydrophobic and amphipathic residues at the interface between the pore and voltage sensing domains of the HERG channel at a region constituting a unique crevice in mammalian channels with a non-swapped topology. Free energy perturbation simulations showed more stable binding in the closed state of the channel suggesting a conformation selection mechanism of ceramide binding, which could explain the inhibitory actions of this lipid (29). However, given the distinctive packing of the VSD against the PD in K_V_1.3 with the canonical swapped domains, such direct Cer binding seems less likely in the case of K_V_1.3. Nevertheless, future studies investigating possible direct Cer- or GlcCer-K_V_1.3 interactions are required to fully elucidate the mechanism of potential direct actions on K_V_ channels.

In general, modification of lipid levels in cellular membranes can be achieved in two different ways. While modulation of the activity of enzymes involved in lipid metabolism using specific inhibitors, activators, or the enzymes themselves is often considered as a more (patho)phyisological method, we opted for loading with exogenous lipids based on the following reasons. i) Our main goal in the current manuscript was to compare the effects of Cer and GlcCer on K_V_1.3 and membrane biophysics. Although there is a well-established, short-term, enzymatic protocol to produce ceramides via the activation of sphingomyelinases (24, 30, 32, 45), no such method has been described yet for glucosylceramides, the most widely used protocol utilizing conduritol B epoxide (CBE), an inhibitor of β-glucocerebrosidase, leads to glucosylceramide accumulation only in case of long-term (typically 72–96 h) exposure (35, 41, 44). Hence, due to the largely different duration of enzyme modulation treatments, results obtained with sphingomyelinases and CBE would not be comparable. ii) When applying exogenous enzymes or modulating the activity of endogenous ones, at least two changes occur: both the levels of the precursor substrate and the product are altered simultaneously, and thus it is always questionable to what extent the altered levels of the substrate and the product contribute to the observed effects. This shortcoming would be especially outstanding for CBE treatment, which would increase the concentration of one of the lipid species we studied (GlcCer) while decreasing that of the other (Cer). Furthermore, modulatory actions of bacterial sphingomyelinase have been described on K_V_1.3 channels expressed in *Xenopus* oocytes, however, these have been attributed to reduced sphingomyelin levels rather than an elevation in ceramide content (32). iii) Enzymatic treatments such as sphingomyelinases or CBE are generally not chain length specific, and considering the significantly different effects of ceramides of varying chain lengths (60), interpretation of the results would be more difficult.

In our study, we loaded cells with exogenous lipids for 24 h at the highest concentration not compromising cell viability (supplemental Fig. S1), which was previously used efficiently in other studies (46, 47, 48). Our loading protocol to elevate ceramide levels was successful as demonstrated by i) a dose-dependent reduction in cell viability at higher concentrations (supplemental Fig. S1) consistent with previous studies demonstrating ceramide-induced cell death in response to large increases in ceramide levels (62, 69); and ii) our experiments to quantify plasma membrane ceramide and glucosylceramide levels using flow cytometry and anti-ceramide or anti-glucosylceramide antibodies, which showed significantly higher ceramide and glucosylceramide levels in response to Cer and GlcCer loading, respectively (supplemental Fig. S2). We applied the lipid treatments for 24 h since i) a 24-h treatment resulted in significantly larger elevations than a 1-h treatment (supplemental Fig. S2); and ii) as opposed to a shorter incubation, a longer treatment may result in an equilibrium of membrane trafficking, while the measurement times that are relatively long in comparison with treatment duration in the case of a 1-h loading would hamper data interpretation in this aspect. However, the production of lipid metabolites might take place even when loading exogenous lipids, and the unknown contribution of this phenomenon to the observed effects might interfere with the conclusions of our study. While this indeed has to be taken into account when interpreting our results, in our opinion such substantial, confounding effects can be ruled out since (i) our findings are in good agreement with previous studies examining ceramide effects on K_V_1.3 and other K_V_ channels (24, 25, 26, 27, 28, 29, 30, 31); (ii) Cer treatment resulted in a dipole potential lowering effect (Fig. 7), while the opposite is expected from other sphingolipids; (iii) due to the strong interconnectivity between ceramide and glucosylceramide metabolic pathways (69), accumulation of a common metabolite should take place if significant, compensatory metabolic pathways were activated, and therefore similar changes should be seen after Cer and GlcCer, however, we observed contrasting effects both in K_V_1.3 function and membrane biophysics; and iv) we detected no notable ceramide accumulation in response to GlcCer loading, and no changes in glucosylceramide levels after Cer loading even in the case of 24-h treatments (supplemental Fig. S2).

At first glance, the possibility to exogenously load cells with Cer containing a long C16-chain seems counterintuitive due to its extreme hydrophobicity resulting in practical insolubility in aqueous media (62). Nevertheless, treatment protocols consistent with ours have been widely reported to successfully modulate membrane ceramide levels (46, 47, 48). While description of the exact mechanism of cellular long-chain ceramide uptake is beyond the scope of the current manuscript, it can be expected that such ceramides might be able to get into cells via different, more complex means than truly water-soluble short-chain (C2 or C6) species. It is conceivable that insoluble ceramide particles formed in an aqueous medium would settle onto the surface of cells and get endocytosed especially in the case of a long-term incubation. One could argue that this would lead to the eventual degradation of ceramides into sphingosine backbone and, consequently, to the large-scale production of various sphingolipid species other than ceramide, which could question the conclusions of our study. Although this scenario may not be completely ruled out, several lines of evidence discussed in the previous paragraph convincingly show that membrane ceramide levels are indeed most predominantly elevated in response to Cer loading. This can be explained by the fact that (i) ceramide particles could evade such degradation, or (ii) the increased abundance of the sphingosine backbone resulting from the exogenous loading may in turn activate the endogenous ceramide production as suggested earlier (70). While due to the solubility problem, the expected effective concentration of Cer during our treatment is definitely below the applied 40 μM, our results show that membrane ceramide levels are significantly elevated in response to our treatment and the conclusions of our study regarding ceramide effects should be valid even without knowing the exact concentration.

Based on our TEVCF measurements, ceramide modifies the VSD movements (Figs. 2 and 4) eventually leading to current inhibition (Fig. 5). Simultaneously, as shown by changes in membrane biophysical properties, this is associated with massive structural reorganization at the membrane-water interface (Fig. 7). What can be the link between the two phenomena, what could be the specific mechanism of ceramide actions? In response to depolarization, the positive gating charges of the S4 helix move outwards from the plane of the membrane during activation gating (Fig. 1) resulting in a complex conformational change in the VSD and consequently in the PD eventually leading to the flow of ions. In our experiments, ceramide shifted the V_1/2_ value of F-V curves (Fig. 2C), which implied an increase in the free energy barrier during gating, while the slope factor of F-V curves remained unaffected (Fig. 2E) suggesting that the effective number of gating charges stayed the same. An increase in the activation time constant of the fluorescence signal refers to a decelerated VSD motion (Fig. 4). These changes can be explained by a model according to which ceramide induces a significant increase in molecular ordering in the uppermost layer of the membrane (Fig. 7) in which the main initial VSD rearrangement involving an outward sliding of the top of S4 occurs (Fig. 1). These alterations block the movement of the VSD and culminate in an inhibition of channel conductance. In accordance, in the Shaker V478W mutant, besides similar changes in the G-V curve, reduced ionic and gating currents, and rightward shifts were reported in the Q (gating charge)-V curve with no changes in its slope factor in response to sphingomyelinase C treatment that also elevated ceramide levels (32). As proposed there, the loss of phosphate groups during the conversion of sphingomyelin to ceramide leads to the loss of countercharges that would otherwise facilitate the movement of gating charges. This does not rule out our model, as the appearance of ceramide is also associated with significant membrane biophysical effects and the two factors may contribute together to the effect of ceramide on the VSD. One could also speculate that ceramide acts primarily through the pore, which would be transmitted back to the VSD in a reverse coupling process since the movement of the activation gate during pore opening occurs at the same, outermost membrane layer, just on the intracellular side. However, this scenario is unlikely because previous single-channel measurements have shown no changes in the unitary conductance, only a decrease in the open probability of the channel in response to ceramide, which also suggests a primary involvement of the VSD instead of the pore (31). On the other hand, changes in the molecular arrangement at the membrane-water interface induced by GlcCer, albeit presumably acting in the same direction, may not be large enough to modify VSD movements so that this lipid affects only the pore opening of K_V_1.3.

The biological relevance of our results stems from the fact that in many diseases ceramide and glucosylceramide levels are changed, which may significantly influence the operation of ion channels, contributing to the pathomechanism of these lipids. For example, elevated amounts of ceramides and their pathophysiological relevance are widely described in cardiometabolic and neurodegenerative disorders (12, 13, 14), while ceramide levels and their production are reduced in tumors contributing to apoptosis resistance (39, 71). On the other hand, glucosylceramides are pathognomically accumulated in Gaucher and Parkinson's diseases and can be involved in the multidrug resistance of tumors (10, 11, 69). Besides their relevance in pathological conditions, these lipids have essential physiological roles as well. Ceramides are typically found at very low levels in the cell membrane, but certain stress signals including tumor necrosis factor α (TNFα), ionizing radiation, and chemotherapeutic drugs largely increase their abundance, which facilitates signaling mechanisms leading to different forms of cell death, autophagy, mitophagy, cytoskeleton rearrangement and senescence (62, 69). In contrast, glucosylceramides are rather broadly present in cellular membranes and are involved in the lateral organization of bilayers (41, 72). Given that K_V_ channels are linked to practically all of the above-described pathological conditions and cellular processes, elucidating the link between altered lipid levels and the consequent ionic current effects and their mechanisms in K_V_ channels would help in the better understanding of these disorders and aid the development of novel therapeutic approaches.

## Data availability

The data that support the findings of this study are available from the corresponding author upon reasonable request.

## Supplemental data

This article contains supplemental data.

## Conflict of interest

The authors declare that they have no conflicts of interest with the contents of this article.
