# Supplementary material for: Novel insights into the modulation of the voltage-gated potassium channel KV1.3 activation gating by membrane ceramides
Source: J Lipid Res. 2024 Jul 15;65(8):100596. doi: 10.1016/j.jlr.2024.100596 (PMC11367112; doi:10.1016/j.jlr.2024.100596)
Supplement: Supplementary material [file mmc1.docx]

# Supplementary methods

Cell viability measurements

CHO cells were seeded into 24-well plates at 50.000/well density and allowed to attach for 4 h, which was followed by treatment with dilution series of Cer or GlcCer with concentrations ranging from 5 to 160 µM for 24 h at 37 °C. As a positive control, cells were treated for 1 h with 0.5% Triton X-100. Cells in suspension and those detached by trypsin treatment were washed and labeled with Sytox Green Dead Cell Stain (Thermo Fisher Scientific) and Alexa Fluor 647-conjugated annexin V (Thermo Fisher Scientific) at dilutions of 1:1000 and 1:20, respectively, in annexin binding buffer for 15 minutes at room temperature. Fluorescence intensities of individual cells were subsequently measured using a NovoCyte 3000RYB flow cytometer (ACEA Biosciences, San Diego, CA). Sytox Green and Alexa Fluor 647 fluorophores were excited at 488 and 640 nm, respectively, and emitted intensities were measured using 530/30 and 660/20 emission filters, respectively. During data analysis the fraction of Sytox Green and annexin V negative cells corresponding to living cells was calculated for each sample using FCS Express (De Novo Software, Los Angeles, CA) and subsequently normalized to the mean value determined in untreated, control samples.

Determination of cell membrane ceramide levels

Control cells grown in 24-well plates and those treated with 40 µM Cer or GlcCer for 1 h or 24 h were trypsinized, washed and labeled with anti-ceramide antibodies clone 15B4 (Sigma-Aldrich) for 60 min at room temperature, followed by washing and a 20-min staining with AlexaFluor647-conjugated goat anti-mouse IgM antibody (Thermo Fisher Scientific) at room temperature. Alternatively, for determination of glucosylceramide levels, anti-glucosylceramide (Glycobiotech, Kukels, Germany) and AlexaFluor647-conjugated goat anti-rabbit IgG antibodies (Thermo Fisher Sicentific) were used. After washing, fluorescence intensities of individual cells were measured with NovoCyte 3000RYB using excitation at 640 nm and a 660/20 emission band pass filter. Mean fluorescence intensities of cells with a normal morphology gated on FSC-SSC plots were calculated in each sample in FCS Express, and subsequently normalized to the mean value determined in untreated, control samples.

# Supplementary results

Effects of Cer and GlcCer on cell viability

To test the effects of Cer and GlcCer on cell viability and determine the maximal concentration that does not compromise viability, we treated CHO cells with different concentrations of Cer and GlcCer for 24 h, and determined the fraction of living cells using flow cytometry after labeling necrotic and apoptotic cells with Sytox Green and Alexa Fluor 647-conjugated annexin V, respectively. In our measurements, Cer did not reduce cell viability up to 40 µM concentration, it resulted in significantly lower fraction of double negative, viable cells only at 80 and 160 µM (Supplementary Figure 1). In the contrary, GlcCer increased the fraction of dead cells only at 160 µM, while absolute ethanol, used to solubilize the lipids and applied at concentrations corresponding to those when performing lipid loading, failed to induce any significant effects on the fraction of living cells. Based on these measurements, we applied these lipids at 40 µM in our subsequent experiments investigating alterations in K_V_1.3 electrophysiological parameters and membrane biophysical properties.


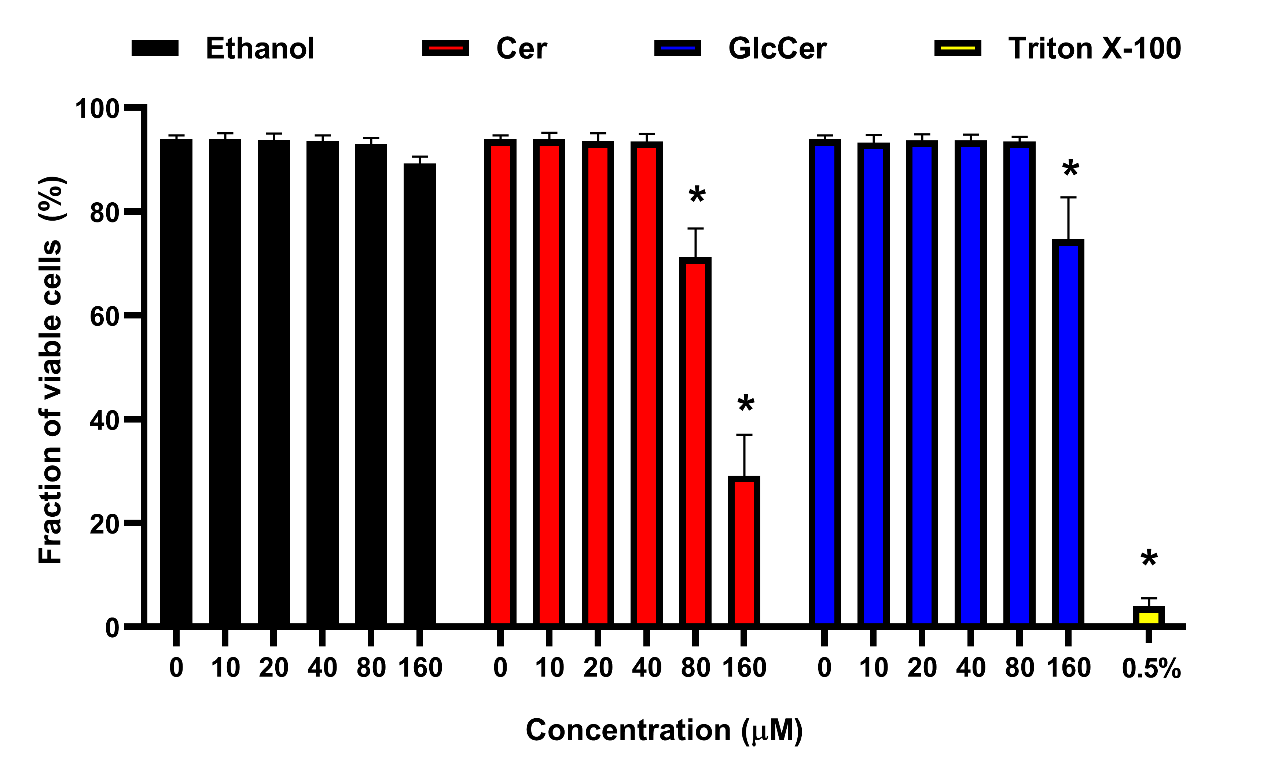


**Supplementary Figure 1.** Effects of Cer and GlcCer on cell viability. Control CHO cells and those treated for 24 h with 10-160 µM Cer or GlcCer, or with the vehicle, ethanol at concentrations corresponding to those used when performing lipid loading were subsequently labeled with Sytox Green and Alexa Fluor 647-conjugated annexin V to identify necrotic and apoptotic cells, respectively. As a positive control, cells were treated for 1 h with 0.5% Triton X-100. Fluorescence intensities of individual cells were measured using flow cytometry and the relative fraction of double negative living cells was determined in each sample containing at least 10,000 cells. The average values (± SEM) obtained from data of n = 5 independent experiments are plotted in the figure as a function of the applied concentration. Asterisks (*) indicate significant differences compared to control samples (p < 0.05, ANOVA followed by Tukey’s HSD test).

Effects of Cer and GlcCer on membrane ceramide and glucosylceramide levels

Although our previous experiments demonstrated that long-term supplementation of the culture medium with Cer (and at extremely high concentrations with GlcCer) affected cell viability and thus expected to incorporate into the plasma membrane of treated cells, we further performed quantitative analysis of membrane ceramide and glucosylceramide levels to further support membrane incorporation of these lipids. We treated CHO cells with 40 µM Cer and GlcCer for 1 or 24 h, and subsequently performed indirect immunofluorescence labeling and flow cytometry to investigate alterations in the amounts of ceramide and glucosylceramide in the plasma membrane in response to the treatments. We found that while Cer loading significantly increased membrane ceramide levels both after 1 and 24 h, GlcCer loading resulted in no significant elevations of ceramide. In the contrary, when examining plasma membrane glucosylceramide levels, GlcCer loading resulted in significant elevations at both time points, while Cer loading induced no significant changes in the amount of glucosylceramide in the plasma membrane. When comparing results obtained after short-term and long-term loading, we found that lipid alterations induced by 24-h loadings were significantly larger than after 1-h incubation both for Cer and GlcCer. These results implied that treatment of cells with exogenous Cer or GlcCer resulted in long-term elevations in plasma membrane Cer and GlcCer levels, respectively, which were superior to those after short-term exposure. Furthermore, the fact that no relevant changes were observed in glucosylceramide levels in response to Cer, and ceramide levels due to GlcCer loading suggest that there was no high-level Cer-GlcCer interconversion and due to the strong connectivity of ceramide-glucosylceramide pathways these argue against activation of compensatory metabolic pathways.


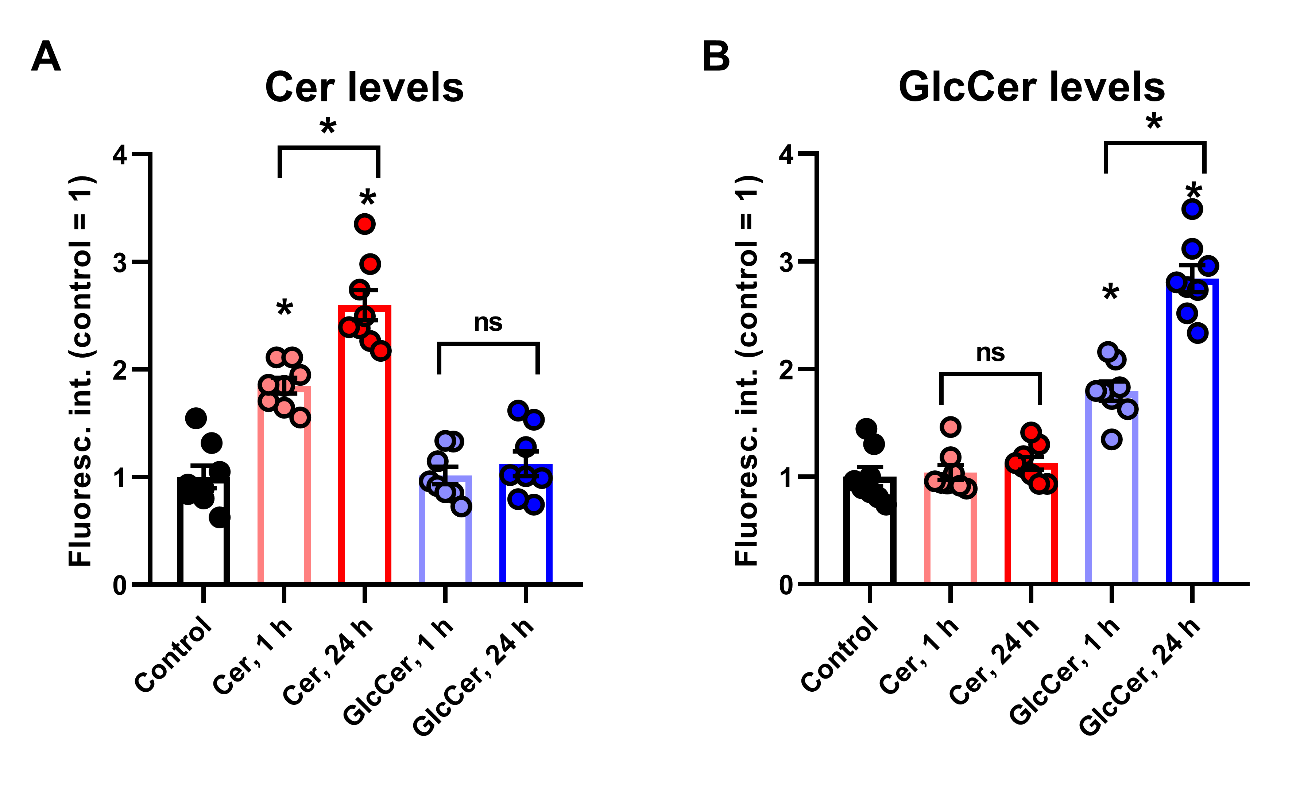


**Supplementary Figure 2.** Effects of Cer and GlcCer on cell membrane ceramide and glucosylceramide levels. Control CHO cells and those treated 40 µM Cer or GlcCer for 1 or 24 h were labeled with anti-ceramide antibodies followed by AlexaFluor647-conjugated goat anti-mouse IgM antibodies (**A**), or anti-glucosylceramide and AlexaFluor647-conjugated goat anti-rabbit IgG antibodies (**B**). Fluorescence intensities of individual cells were measured using flow cytometry and the average fluorescence intensity of at least 10,000 cells of normal morphology per sample was determined and subsequently normalized to the mean value determined in the untreated, control sample. The normalized fluorescence intensity values obtained in n = 8 independent samples, and their average values (± SEM) are plotted in both panels. Asterisks (*) indicate significant differences compared to control samples, or between samples treated for 1 and 24 h (p < 0.05, ANOVA followed by Tukey’s HSD test).
